# Relationship between Information Scrambling and Quantum Darwinism

**DOI:** 10.3390/e26010019

**Published:** 2023-12-24

**Authors:** Feng Tian, Jian Zou, Hai Li, Liping Han, Bin Shao

**Affiliations:** 1 School of Physics, Beijing Institute of Technology, Beijing 100081, China; tianfeng5g@163.com (F.T.); sbin610@bit.edu.cn (B.S.); 2 School of Information and Electronic Engineering, Shandong Technology and Business University, Yantai 264005, China; lihai@sdtbu.edu.cn; 3 School of Science, Tianjin University of Technology, Tianjin 300384, China; hanlp9902@bit.edu.cn

**Keywords:** quantum Darwinism, information scrambling, collision model, quantum mutual information, tripartite mutual information

## Abstract

A quantum system interacting with a multipartite environment can induce redundant encoding of the information of a system into the environment, which is the essence of quantum Darwinism. At the same time, the environment may scramble the initially localized information about the system. Based on a collision model, we mainly investigate the relationship between information scrambling in an environment and the emergence of quantum Darwinism. Our results show that when the mutual information between the system and environmental fragment is a linear increasing function of the fragment size, the tripartite mutual information (TMI) is zero, which can be proved generally beyond the collision model; when the system exhibits Darwinistic behavior, the TMI is positive (i.e., scrambling does not occur); when we see the behavior of an “encoding” environment, the TMI is negative (i.e., scrambling occurs). Additionally, we give a physical explanation for the above results by considering two simple but illustrative examples. Moreover, depending on the nature of system and environment interactions, it is also shown that the single qubit and two-qubit systems behave differently for the emergence of quantum Darwinism, and hence the scrambling, while their relationship is consistent with the above conclusion.

## 1. Introduction

Quantum Darwinism is a theoretical framework that allows one to understand the emergence of objectivity out of quantum superpositions [1]. Due to the interaction between system and environment, the latter acquires information about the state of the former with respect to the so-called pointer states [2,3,4,5], namely, the eigenstates of the observable, which is coupled with the environment [6,7,8,9]. Pointer states are left undisturbed during the interaction with the environment, but if the system is in a coherent superposition of pointer states, it gets entangled with the environment. Many observers can independently access and measure different parts of the environment and independently obtain the same information about the system [10,11,12]. This redundancy is a characteristic feature of quantum Darwinism [13,14,15], explaining the emergence of objective reality.

Quantum Darwinism has been extensively studied in various models [16,17,18,19,20,21,22,23,24,25]. One of the main issues of these investigations is to understand the fundamental mechanism through which quantum Darwinism emerges. Recently, several works have shown that the emergence of quantum Darwinism is sensitive to the microscopic description of quantum dynamics, such as the nature of interaction and initial conditions [26,27]. It has been shown that the nature of correlations among the environmental constituents, i.e., whether they are quantum or classical, is important for the emergence of quantum Darwinism [27]. The authors in Reference [28] have validated that in quantum Darwinism, when classical objectivity manifests, bipartite quantum correlations between the system and components of its environment are suppressed. Experimental investigations of quantum Darwinism were also reported [29,30,31]. Generally, quantum Darwinism needs to analyze quantum mutual information between system and environment; hence, it is necessary to keep track not only of the system but also of the environment. The master equation is widely used to obtain the dynamics of an open system by tracing out environmental degrees of freedom, while it cannot treat the full system–environment dynamics. The quantum collision model (CM), which decomposes complicated dynamics in terms of discrete elementary processes, offers an alternative way of describing open quantum system dynamics [32,33,34,35,36]. Additionally, for CM, the correlations between the open quantum system and its environment can be easily traced. In the standard framework of CM, the environment is represented by an ensemble of uncorrelated identical environment constituents termed ancillas, and the system of interest interacts with each ancilla sequentially. Recently, CMs have found an application in the investigation of quantum Darwinism [26,27,37].

How information spreads and becomes distributed over the constituent degrees of freedom is one of the central issues in the study of dynamics in the quantum many-body system. The delocalization of information in a many-body system is referred to as information scrambling, which has attracted more and more attention [38,39,40,41]. A general accepted measure of information scrambling is the so-called out-of-time-order correlator (OTOC) [41,42,43,44], which is associated with the growth of the square commutator between two initially commuting observables. Besides OTOC, other measures have been proposed, such as the average Pauli weight [45] and the operator entanglement entropy [46]. Beyond these, the tripartite mutual information (TMI) is an important measure of information scrambling [41]. At first, TMI of the evolution operator was used to investigate information scrambling in References [47,48]. Later, instantaneous TMI of a quantum state was also used to study information scrambling in References [49,50,51]. The method used in this paper is the instantaneous TMI of a quantum state.

In terms of TMI, scrambling in the many-body quantum system has been studied in various models [47,50,51,52]. Their typical setting of studying information scrambling in the many-body quantum system by the TMI consists of an ancillary system and a many-body system. At the initial time, the information of the ancillary system is locally encoded in the many-body system through entanglement. The many-body system then evolves unitarily, and the locally encoded information might spread over the entire many-body system. Within such a setting, information scrambling in a central spin model has been studied in Reference [53], where information that initially resides in the central spin is first shared with the environment due to the coupling between them. Then, only the environment dynamics are turned on, causing the scrambling of information in the environment. On the other hand, Reference [54] studied a scenario where a nuclear spin (the system) is simultaneously coupled with a large collection of non-interacting bath spins. In this situation, scrambling of information in the bath is due to the interaction between the central spin and bath. This is different from the typical scenario (a many-body system plus an ancillary system) mentioned above, where the scrambling of information is due to the self-dynamics of the many-body system.

In the quantum Darwinistic picture, the environment that acquires redundant information about the system is composed of many subsystems. Therefore, a full characterization of whether the locally encoded information at the beginning is localized or spreads over the entire many-body environment in the time evolution could be helpful for understanding the mechanism of quantum Darwinism. This raises the question of what the relationship is between information scrambling and quantum Darwinism. To this end, we present a unified framework to link these two seemingly unrelated research topics. Specifically, we consider a system interacting with a multipartite environment, which can be considered as a many-body system. Such a model is the basic setting of quantum Darwinism, while it also allows us to analyze information scrambling in the multipartite environment. In this paper, we study scrambling in terms of the TMI among the system and different parts of the environment. There is a reason behind this choice. A widely used measure of quantum Darwinism is the quantum mutual information between the system and a fragment of the environment, which is the key quantifier in capturing redundant information. On the other hand, quantum mutual information is the key ingredient of the TMI in the study on scrambling. It is these two quantifiers that allow us to establish a relationship between the emergence of quantum Darwinism and information scrambling.

**Figure 1:**
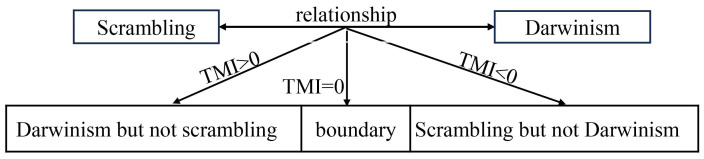
The relationship between information scrambling and quantum Darwinism.

We consider a CM as consisting of a system (one or two qubits) and an environment (a collection of ancillas), which can be considered as a quantum-body system. In our model, similar to Reference [54], the scrambling of initially localized system information in the environment is not due to the self-dynamics of a many-body system or environment, but rather the sequential system–ancilla interactions. We mainly consider the pure dephasing and exchange interactions between the system and environment, respectively. We find that when the system exhibits Darwinistic behavior, the TMI is positive; when we see the behavior of an “encoding” environment, the TMI is negative, namely, scrambling occurs; when the mutual information between the system and environmental fragment is a linear function of fragment size (namely, a boundary between the former two cases), the TMI is zero [see Figure 1]. Additionally, we explained the physical mechanism of these results. Moreover, depending on the nature of interactions between the system and environment, our results also show that the single qubit and two-qubit systems behave differently for the emergence of quantum Darwinism, and hence the scrambling, while their relationship is the same as the above conclusion.

## 2. Preliminaries

### 2.1. Model

**Figure 2:**
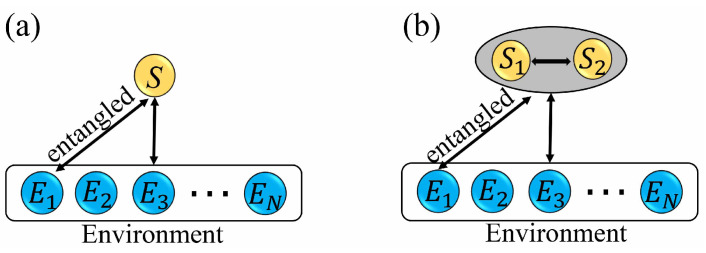
Schematics of the collision model. The system *S* is coupled to an environment *E* composed of a collection of environment ancillas ($E_{1},E_{2},\ldots ,E_{N}$). (**a**) *S* is a single qubit. (**b**) *S* is composed of two interacting qubits $S_{1}$ and $S_{2}$. In both cases, the ancilla $E_{1}$ is initially entangled with *S*, and the remaining ancillas $E_{k}\left(k\geq 2\right)$ are initialized in the same state $|\eta_{k}\rangle$. The system *S* interacts sequentially with the ancillas $E_{1}$, $E_{2}$, …, $E_{N}$.

In this section, we consider a collision model, which consists of a system *S* and an environment *E*. The environment consists of a collection of *N* non-interacting environment ancillas $\left(E_{1},E_{2},\ldots ,E_{N}\right)$, which can be considered as a many-body system. We consider two scenarios where the system *S* contains a single qubit (Figure 2a) and two qubits (Figure 2b), respectively. For the single qubit system, the Hamiltonian is given by

(1) $$H_{S}^{1}=\frac{1}{2}\sigma_{z}$$

with Pauli operator $\sigma_{z}$ (we set ℏ=1). For the two-qubit system, the Hamiltonian is

(2) $$H_{S}^{2}=H_{S_{1}}+H_{S_{2}}+H_{S_{1},S_{2}},$$

where $H_{S_{i}}=\frac{1}{2}\sigma_{z}\;\left(i=1,2\right)$ is the free Hamiltonian of $S_{i}$, and $H_{S_{1},S_{2}}$ is the interaction Hamiltonian between $S_{1}$ and $S_{2}$. We take $H_{S_{1},S_{2}}$ as

(3) $$H_{S_{1},S_{2}}=\epsilon \left(\sigma_{x}⊗\sigma_{x}+\sigma_{y}⊗\sigma_{y}\right)$$

with coupling strength ϵ. We assume that each ancilla of the environment is a qubit, and its Hamiltonian is given by $H_{E_{k}}=\frac{1}{2}\sigma_{z}$.

Initially, the information of the system is locally encoded in the environment by entangling the system and the first ancilla $E_{1}$ in the environment. Specifically, we set the initial state of the system and environmental ancillas to be:

(4) $$|\psi_{0}\rangle =|\phi_{SE_{1}}\rangle \overset{N}{\underset{k=2}{⨂}}|\eta_{k}\rangle ,$$

where $|\phi_{SE_{1}}\rangle$ is an entangled state between the system and ancilla $E_{1}$, and $|\eta_{k}\rangle$ is the initial state of the *k*th (k≥2) ancilla $E_{k}$. The evolution of the whole system is described in terms of pairwise short interactions between *S* and each environment ancilla: *S* first collides with $E_{1}$; then, *S* collides with $E_{2}$, and so on. In this way, at each step, *S* collides with a fresh ancilla. A schematic sketch of the collision model is given in Figure 2.

We consider the general Heisenberg interaction between the system and environment ancilla. For the single qubit system, the system–ancilla interaction Hamiltonian is given by

(5) $$H_{S,E_{k}}^{1}=\sum_{j=x,y,z}J_{j}\left(\sigma_{S}^{j}⊗\sigma_{E_{k}}^{j}\right)$$

with coupling strength $J_{j}\;\left(j=x,y,z\right)$. For the two-qubit system, the system–ancilla interaction Hamiltonian is given by

(6) $$H_{S,E_{k}}^{2}=\sum_{j=x,y,z}J_{j}\left(\sigma_{S_{1}}^{j}⊗\sigma_{E_{k}}^{j}+\sigma_{S_{2}}^{j}⊗\sigma_{E_{k}}^{j}\right).$$

For the single qubit system, the interaction between the system *S* and ancilla $E_{k}$ in any *k*th step is realized by the application of the unitary operation

(7) $$U_{S,E_{k}}^{1}=\exp\left[-i\left(H_{0}^{1}+H_{SE_{k}}^{1}\right)t\right],$$

where $H_{0}^{1}=H_{S}^{1}+H_{E_{k}}$, and *t* stands for the interaction time, i.e., the duration of each collision. Similarly, for the two-qubit system, the interaction between the system *S* and ancilla $E_{k}$ in any *k*th step is realized by

(8) $$U_{S,E_{k}}^{2}=\exp\left[-i\left(H_{0}^{2}+H_{SE_{k}}^{2}\right)t\right]$$

with $H_{0}^{2}=H_{S}^{2}+H_{E_{k}}$. Thus, for the single qubit system, the initial joint syste-m-environment state $\rho_{0}^{1}$, after *N* steps, evolves into

(9) $$\rho_{N}^{1}=U_{\left\{N\right\}}^{1}\rho_{0}^{1}U_{\left\{N\right\}}^{1†}$$

with $U_{\left\{N\right\}}^{1}=U_{S,E_{N}}^{1}U_{S,E_{N-1}}^{1}\ldots U_{S,E_{2}}^{1}U_{S,E_{1}}^{1}$. Similarly, for the two-qubit system, the initial joint system–environment state $\rho_{0}^{2}$, after *N* steps, evolves into

(10) $$\rho_{N}^{2}=U_{\left\{N\right\}}^{2}\rho_{0}^{2}U_{\left\{N\right\}}^{2†}$$

with $U_{\left\{N\right\}}^{2}=U_{S,E_{N}}^{2}U_{S,E_{N-1}}^{2}\ldots U_{S,E_{2}}^{2}U_{S,E_{1}}^{2}$.

### 2.2. Information Scrambling

**Figure 3:**
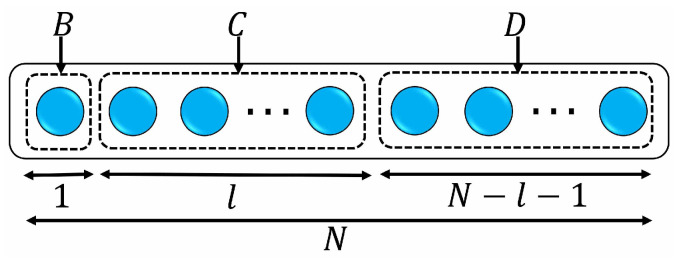
The whole environment *E* is divided into three nonoverlapping subsystems *B*, *C*, *D*, whose sizes (the numbers of the ancillas) are, respectively, given by 1, *l*, and N−l−1.

For the CM we considered in Figure 2, at the initial time, the system information is locally encoded in the environment through $S-E_{1}$ entanglement, and then this information might be scrambled as the system interacts sequentially with environmental ancillas. Here, we employ the tripartite mutual information as a quantifier of information scrambling [41,49,50,51]. As shown in Figure 3, we divide the whole environment *E* into three nonoverlapping subsystems *B*, *C*, *D*, whose sizes (the numbers of the ancillas) are, respectively, given by 1, *l*, and N−l−1. The tripartite mutual information among three subsystems *S*, *B*, and *C* is defined as

(11) $$I_{3}\left(S:B:C\right)=I\left(S:B\right)+I\left(S:C\right)-I\left(S:BC\right).$$

Here, I(S:X) is the quantum mutual information between *S* and *X* (X=B,C,BC), which quantifies the correlations between two subsystems of a composite system. I(S:X) is defined by

(12) $$I\left(S:X\right)=H_{S}+H_{X}-H_{SX},$$

where $H_{Y}$ = −Tr$\left[\rho_{Y}\ln\rho_{Y}\right]$ is the von Neumann entropy for the reduced state $\rho_{Y}$ of subsystem *Y*. From an information-theoretic point of view, TMI quantifies how the total (quantum and classical) information is shared among the subsystems *A*, *B*, and *C*. Unlike mutual information, TMI has no definite sign. TMI is negative when I(S:B)+I(S:C)<I(S:BC), which implies that information about *S* stored in composite BC is larger than the sum of the amounts of information that *B* and *C* have individually. In this case, the information about *S* is nonlocally stored in *B* and *C* such that measurements on *B* and *C* alone are not able to reconstruct *S*. When TMI is non-negative at some time, the information at this moment is localized, while at some time when TMI is negative, the information is delocalized. If TMI is non-negative at the beginning and becomes negative as time evolves, the information is gradually delocalized, namely, information scrambling occurs. It is noted that for a given *l*, there are many partitions of environment when dividing the whole environment *E* into three nonoverlapping subsystems *B*, *C*, *D*. Therefore, we compute the averaged TMI ${\bar{I}}_{3}\left(S:B:C\right)$ defined as the averaging over all possible partitions of *E* for a given *l*.

### 2.3. Quantum Darwinism

**Figure 4:**
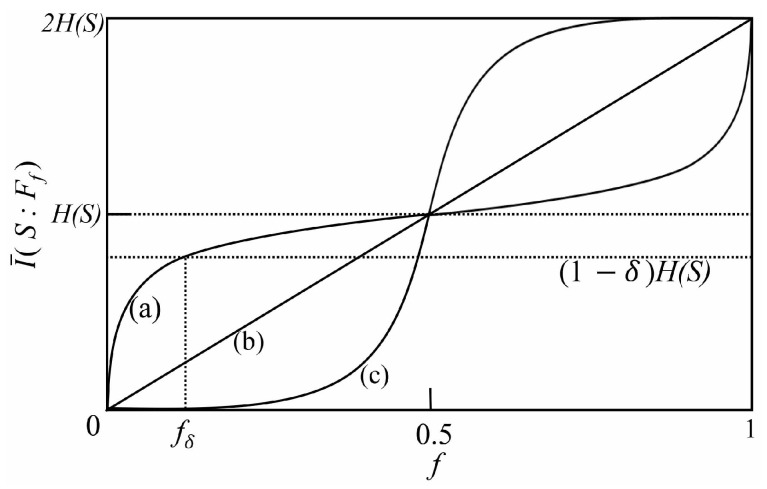
$\bar{I}\left(S:F_{f}\right)$ as a function of *f*. Line (a) corresponds to the redundancy of Darwinism, where a small fraction $f_{\delta }$ of *E* contains almost all (but δ) of the information about *S*. The linear profile (b) shows the behavior of an “independent” environment. Line (c) shows an “encoding” environment or an antiredundancy, i.e., $\bar{I}\left(S:F_{f}\right)$ takes on an *S*-shaped profile.

In quantum Darwinism, the signature of objectivity is described by the quantum mutual information between the system *S* and a fragment of the environment *E*. Any individual environment fragment $F_{f}\subseteq E$ contains fN individual subsystems if *E* is compoesd of *N* individual quantum systems. Here, f∈[0,1] is the fraction of *E* contained in $F_{f}$. According to Equation (12), the mutual information between *S* and $F_{f}$ is

(13) $$I\left(S:F_{f}\right)=H_{S}+H_{F_{f}}-H_{SF_{f}},$$

where $H_{S}$, $H_{F_{f}}$, and $H_{SF_{f}}$ are the von-Neumann entropies of *S*, $F_{f}$, and $S+F_{f}$, respectively. Since there are many fragments of a given size *f*, the averaged mutual information $\bar{I}\left(S:F_{f}\right)$ is defined by the average of $I(S:F_{f})$ over all possible fragments $F_{f}$. The emergence of redundant information is encoded throughout the environment, and therefore, the quantum Darwinism is detected by the existence of the smallest fragment size $f_{\delta }$ such that $\bar{I}\left(S:F_{f_{\delta }}\right)\geq \left(1-\delta \right)H_{S}$, i.e., when the smallest fragment $F_{f_{\delta }}$ contains roughly all (but δ) the information of the system state. Here, the information deficit, δ∈(0,1), is the information that observers are prepared to forgo. In a plot of $\bar{I}\left(S:F_{f}\right)$ versus fragment size *f*, such redundancy features are characterized by a rapid rise of $\bar{I}$ at relatively small *f*, followed by a long “classical plateau” (see line (a) in Figure 4). In the plateau region, as fragment size *f* increases, $F_{f}$ provides roughly the same information about the system. $\bar{I}$ increases to $2H_{S}$ only when the fragment encompasses the whole environment, i.e., f=1. Moreover, the curve ($\bar{I}$ vs. *f*) can take two other basic shapes [14,15]: the linear profile (see line (b) in Figure 4) corresponds to the behavior of an “independent” environment, where each subenvironment provides unique and independent information about *S*; the *S*-shaped profile corresponds to the behavior of an “encoding” environment (see line (c) in Figure 4), where information about *S* is encoded in multiple subenvironments, and to learn about the system, one requires access to at least half of the environment. For a pure state of the whole system and environment, the curve ($\bar{I}$ vs. *f*) is always antisymmetric with respect to $\bar{I}=H_{S}$ at f=0.5 [15].

## 3. Results

In this section, we present our numerical results based on the above CM. We are interested in whether or not the system information initially locally encoded in an environment is scrambled as time evolves, whether or not such dynamics can induce the emergence of quantum Darwinism, as well as the relationship between these two phenomena. Different choices of $J_{j}$ in Equations (5) and (6) create different system–environment interactions. We will consider two types of system–environment interactions, the pure dephasing interaction and exchange interaction, for both the single qubit and two-qubit systems, repectively.

### 3.1. Dephasing Channel

Now, we consider the pure dephasing channel, i.e., $J_{x}=J_{y}=0$ and $J_{z}=J$. For the single qubit system, the initial entangled state $|\phi_{SE_{1}}\rangle$ between the system and ancilla $E_{1}$ is supposed to be prepared in

(14) $$|\phi_{SE_{1}}{\rangle =(|-\rangle }_{S}{⊗|+\rangle }_{E_{1}}{+|+\rangle }_{S}{⊗|-\rangle }_{E_{1}})/\sqrt{2}$$

with ${|\pm \rangle }_{S(E_{1})}=(|0\rangle \pm \left|1\rangle \right)/\sqrt{2}$. All the ancillas $E_{k}$ (k≥2) are initially prepared in the identical state $|\eta_{k}\rangle =(|0\rangle +|1\rangle )/\sqrt{2}$.

**Figure 5:**
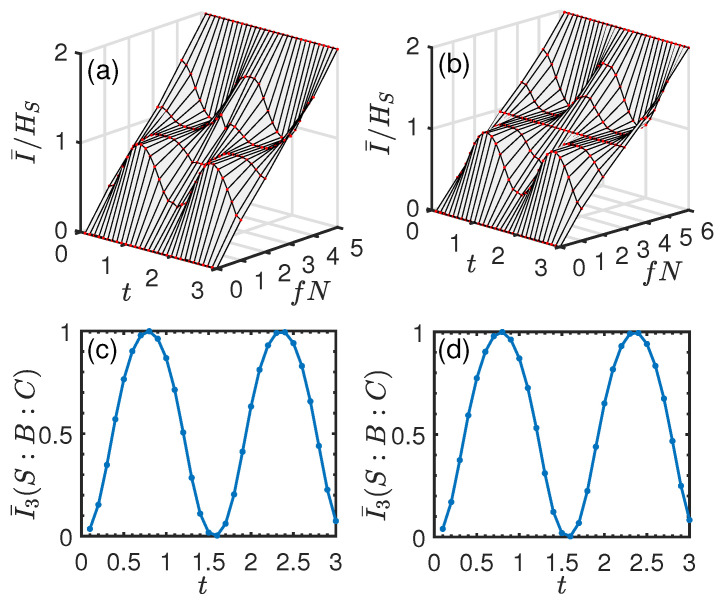
Normalized mutual information $\bar{I}/H_{S}$ and averaged TMI ${\bar{I}}_{3}$ for the pure dephasing system–environment interaction in the case of a single qubit system. Upper panel: $\bar{I}/H_{S}$ vs. fN and *t*. Lower panel: ${\bar{I}}_{3}\left(S:B:C\right)$ as a function of *t*. (**a**,**c**) N=5. (**b**,**d**) N=6. We set J=1 and l=2. $\bar{I}/H_{S}$ is plotted by averaging over 1000 possible environment fragments for the same *f*, and ${\bar{I}}_{3}$ is plotted by averaging over 1000 possible environment partitions, respectively.

**Figure 6:**
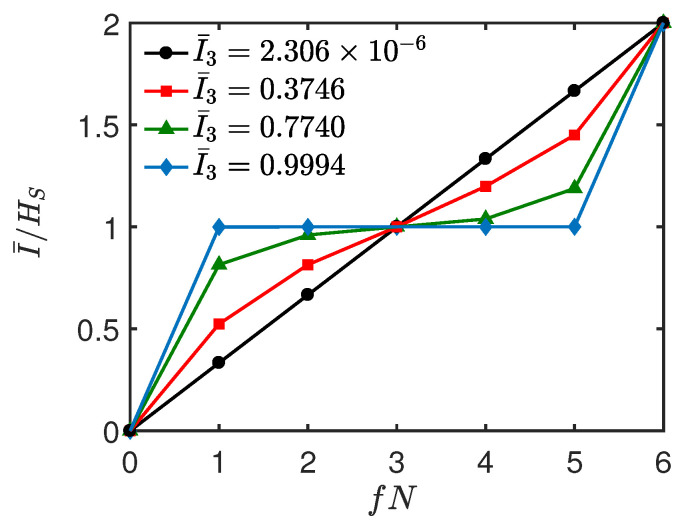
Normalized mutual information $\bar{I}/H_{S}$ as a function of fN at different *t*. The red (squares), green (triangles), blue (diamonds), and black (circles) lines correspond to *t* = 0.3, 0.5, 0.8, and 1.59, respectively. All the parameters are the same as those in Figure 5b.

In Figure 5a,b, we show the normalized mutual information $\bar{I}/H_{S}$ versus time *t* and environment fraction fN. In Figure 5c,d, we plot the corresponding averaged ${\bar{I}}_{3}\left(S:B:C\right)$ as a function of *t*, respectively. It can be seen from Figure 5a,b that as *t* increases from 0, the redundancy plateau begins to emerge, and it becomes more and more pronounced with the increase of *t*. Then, this clear signature of objectivity is gradually lost and emerges periodically as *t* increases further. Interestingly, we find that as time evolves, the dynamics behavior of $\bar{I}/H_{S}$ is closely related to that of ${\bar{I}}_{3}$. From Figure 5c,d, it can be seen that ${\bar{I}}_{3}$ is positive, and it experiences successive increasing and decreasing behaviors with the increase of *t*. Specifically, by comparing Figure 5a,c, or Figure 5b,d, it is clear that as ${\bar{I}}_{3}$ increases, the redundancy plateau becomes more and more pronounced and vice versa. To clearly show the relationship between them, in Figure 6, we plot $\bar{I}/H_{S}$ as a function of fN at some instants of time corresponding to different values of ${\bar{I}}_{3}$. From Figure 6, we find that: when $\bar{I}/H_{S}$ is approximately a linear function of fN, which indicates an “independent” environment, the corresponding ${\bar{I}}_{3}\approx 0$ (black, circles line); when ${\bar{I}}_{3}$ increases from 0, the Darwinistic behavior begins to appear, and it becomes more and more pronounced as ${\bar{I}}_{3}$ increases further (e.g., ${\bar{I}}_{3}=0.3746,\;0.7740$); when ${\bar{I}}_{3}=0.9994$, a perfect redundant encoding is observed (blue, diamond line) in the sense that even a single ancilla is sufficient to give all the information about *S*, i.e., quantum Darwinism emerges perfectly.

Physically, the above phenomenon can be understood as follows. Consider a general model beyond CM, which is composed of a quantum system *S* interacting with an environment *E* consisting of a collection of *N* ancillas ($E_{1}$, $E_{2}$, …, $E_{N}$). Here, we do not have any restrictions on the system-environment dynamics. For this general model, we consider the following two cases separately. First, we consider a situation in which the mutual information $I(S:F_{f})$ is a linear function of fragment size *f*, namely, it exhibits a linear profile like line (b) in Figure 4. In this case, we can easily prove that it is related to zero TMI. Specifically, the linear relationship between $I(S:F_{f})$ and *f* means that $I(S:F_{f})$ is proportional to the number of ancillas contained in $F_{f}$, i.e.,

$$I(S:F_{f})=k\cdot fN,$$

where *k* is a constant. According to Equation (11), we have

$$\begin{array}{cc} I_{3}\left(S:B:C\right) & =I(S:B)+I(S:C)-I(S:BC)\\ & =k\cdot 1+k\cdot l-k\cdot (1+l)\\ & =0, \end{array}$$

which is independent of the choice of *l*. Then, we consider the case $I_{3}\left(S:B:C\right)>0$. Positive TMI indicates that more information about the system *S* is shared among individual environmental ancillas. As positive TMI increases, more and more system information will be shared. Therefore, the measurement on a small fraction of the environment can have access to almost the same amount of information about the system, leading to the emergence of quantum Darwinism. Assume that in this general model, at the initial time, the joint system–environment state takes the form

(15) $$|\Psi_{0}\rangle =|\phi_{SE_{1}}\rangle ⊗|\eta_{0}^{2}\rangle ⊗|\eta_{0}^{3}\rangle ⊗\ldots |\eta_{0}^{N}\rangle .$$

Here, $|\phi_{SE_{1}}\rangle$ is an entangled state between the system and ancilla $E_{1}$, and $|\eta_{0}^{j}\rangle$(j=2,…,N) is the initial state of $E_{j}$, respectively. Depending on different system–environment dynamics, the joint system–environment state after the evolution is also different. We consider a situation where the composite system, after time *t*, evolves into the state,

(16) $$|\Psi_{t}\rangle =\sum_{k}\alpha_{k}|\phi_{k}\rangle ⊗|\eta_{k}^{1}\rangle ⊗|\eta_{k}^{2}\rangle ⊗\ldots |\eta_{k}^{N}\rangle ,$$

where $|\phi_{k}\rangle$ is the pointer states of the system, and $\left\{|\eta_{k}^{j}\rangle \right\}$ is the eigenbasis of the ancilla $E_{j}$. We first consider the TMI among the *S*, *B*, and *C*. In terms of Equation (16), tracing out all the environmental ancillas, we obtain the reduced density matrix of the system *S*

(17) $$\rho_{S}=\sum_{k}|\alpha_{k}{|}^{2}|\phi_{k}\rangle \langle \phi_{k}|.$$

Similarly, we can obtain

(18) $$\rho_{B(C)}=\sum_{k}|\alpha_{k}{|}^{2}|\varphi_{k}^{B(C)}\rangle \langle \varphi_{k}^{B(C)}|,$$

(19) $$\rho_{SB(SC)}=\sum_{k}|\alpha_{k}{|}^{2}|\phi_{k}\rangle \langle \phi_{k}\left|⊗\right|\varphi_{k}^{B(C)}\rangle \langle \varphi_{k}^{B(C)}|,$$

where $\varphi_{k}^{B}:={⊗}_{j\in B}|\eta_{k}^{j}\rangle$ and $\varphi_{k}^{C}:={⊗}_{j\in C}|\eta_{k}^{j}\rangle$. Using the definition of Equation (11), we have

(20) $$I_{3}\left(S:B:C\right)=-\sum_{k}{\left|\alpha_{k}\right|}^{2}\log_{2}p_{k}>0.$$

Meanwhile, according to Equation (13), the mutual information between *S* and *B*(*C*) can be obtained as

(21) $$I\left(S:B\right)=I\left(S:C\right)=H\left(S\right)=-\sum_{k}|\alpha_{k}{|}^{2}\log_{2}{\left|\alpha_{k}\right|}^{2},$$

which is independent of *l*. This means that for the joint system and environment state of Equation (16), one can acquire information about the system through the measurement on any size of the fraction of the environment. In other words, the state of Equation (16) is an example of perfect quantum Darwinism in the sense that even a single environmental ancilla is sufficient to access all the information about *S*. Therefore, positive TMI is related to the emergence of Darwinism.

**Figure 7:**
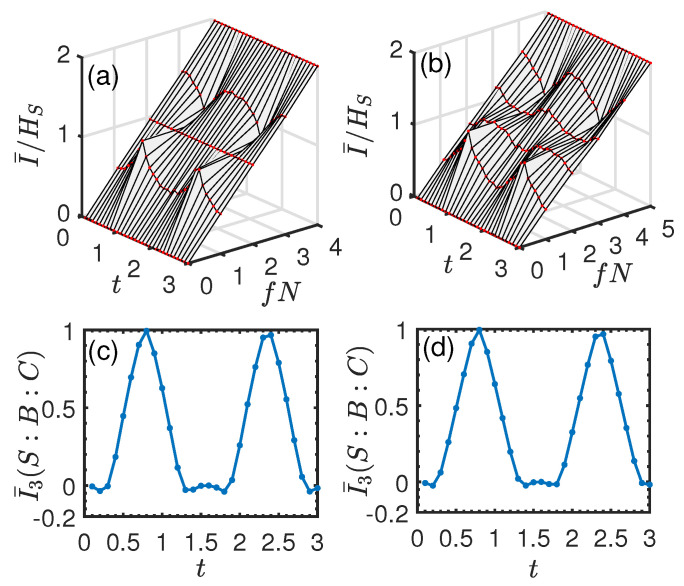
Normalized mutual information $\bar{I}/H_{S}$ and averaged TMI ${\bar{I}}_{3}$ for the pure dephasing system–environment interaction in the case of the two-qubit system. Upper panel: $\bar{I}/H_{S}$ vs. fN and *t*. Lower panel: ${\bar{I}}_{3}\left(S:B:C\right)$ as a function of *t*. (**a**,**c**) N=4. (**b**,**d**) N=5. We set ϵ=1, and the remaining parameters are the same as those in Figure 5.

For the two-qubit system, the initial entangled state $|\phi_{SE_{1}}\rangle$ between the system and ancilla $E_{1}$ is supposed to be prepared in

(22) $${(|-+\rangle }_{S}{⊗|-\rangle }_{E_{1}}{+|+-\rangle }_{S}{⊗|+\rangle }_{E_{1}})/\sqrt{2},$$

and all the ancillas $E_{k}$ (k≥2) are initially prepared in the identical state $|\eta_{k}\rangle =(|0\rangle +|1\rangle )/\sqrt{2}$. Figure 7a,b shows the normalized mutual information $\bar{I}/H_{S}$ as a function of *t* and fN. In this case, its behaviors are qualitatively the same as those of the single qubit system. We also see that a redundancy plateau emerges periodically with the increase of *t*. In Figure 7c,d, we plot ${\bar{I}}_{3}$ as a function of *t*. From these numerical calculations, we again find that: when $\bar{I}/H_{S}$ varies linearly with fN, the corresponding ${\bar{I}}_{3}\approx 0$; as ${\bar{I}}_{3}$ increases from 0, the Darwinistic behavior gradually emerges, and it becomes more and more pronounced as ${\bar{I}}_{3}$ increases further and vice versa.

**Figure 8:**
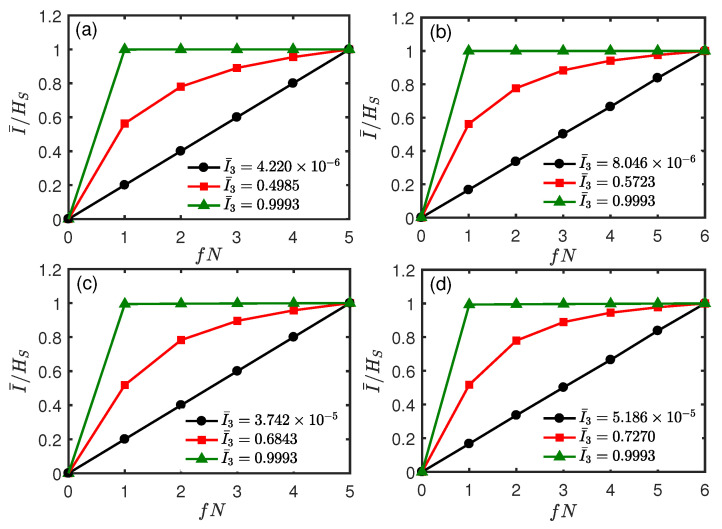
Normalized mutual information $\bar{I}/H_{S}$ as a function of fN for the pure dephasing system–environment interaction. Upper panel: single qubit system with the same parameters except *N* as in Figure 5. Lower panel: two-qubit system with the same parameters except *N* as in Figure 7. The red (squares), green (triangles), and black (circles) lines correspond to *t* = 0.046, 0.80, and 1.20, respectively. (**a**,**c**) N=10. (**b**,**d**) N=12.

Until now, we have only considered the environment *E* consisting of up to N=6 ancillas. From a numerical calculation, in Figure 8, we show that the above results persist for a larger environment N=10 and N=12. Since the global S+E state is pure, the plots ($\bar{I}/H_{S}$ vs. *f*) are antisymmetric about f=1/2. In Figure 8, we plot $\bar{I}/H_{S}$ as a function of fN varying from 0 to N/2. It can be seen that the linear dependence of the mutual information $\bar{I}/H_{S}$ on the environment fraction fN corresponds to the case where ${\bar{I}}_{3}\approx 0$. Additionally, the more pronounced the Darwinistic plateau becomes, the larger the positive ${\bar{I}}_{3}$ is and vice versa, showing that the above results are valid for larger environments.

### 3.2. Exchange Interaction

**Figure 9:**
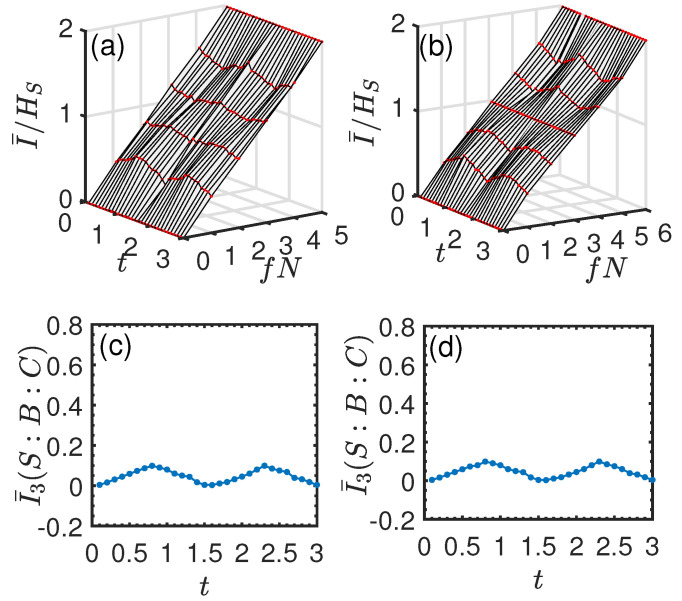
Normalized mutual information $\bar{I}/H_{S}$ and averaged TMI ${\bar{I}}_{3}$ for the exchange system–environment interaction in the case of a single qubit system. Upper panel: $\bar{I}/H_{S}$ vs. fN and *t*. Lower panel: ${\bar{I}}_{3}\left(S:B:C\right)$ as a function of *t*. (**a**,**c**) N=5. (**b**,**d**) N=6. The remaining parameters are the same as those in Figure 5.

Now, we turn our attention to the exchange interaction, i.e., $J_{x}=J_{y}=J$ and $J_{z}=0$. First, we consider the single qubit system. The initial entangled state $|\phi_{SE_{1}}\rangle$ between the system and ancilla $E_{1}$ is initially prepared in the same state as Equation (14) in Section 3.1, and all the ancillas $E_{k}$ (k≥2) are initially prepared in the identical state $|\eta_{k}\rangle =|0\rangle$. In Figure 9a,b, we show the normalized mutual information $\bar{I}/H_{S}$ as a function of fN and *t*. In this case, there is no perfect redundant encoding compared with that of the pure dephasing interaction. Nonetheless, the key features of quantum Darwinism still persist, i.e., a less manifest redundancy plateau appears at some specific time. In Figure 9c,d, we plot ${\bar{I}}_{3}$ as a function of *t*. It can be seen from Figure 9c,d that although ${\bar{I}}_{3}$ is positive, it is much smaller compared with that in Figure 5c,d for all times. This indicates that less information about the system *S* is shared among individual environment ancillas. Thus, the measurement on a small fraction of the environment can only have access to less information about the system. This is why the redundancy plateau is less manifest in this case. Thus, again, the result is consistent with the conclusion in Section 3.1.

**Figure 10:**
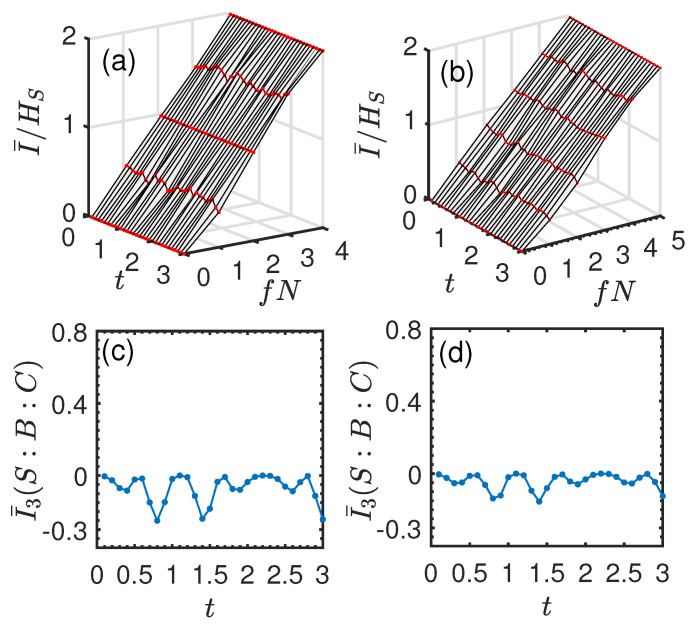
Normalized mutual information $\bar{I}/H_{S}$ and averaged TMI ${\bar{I}}_{3}$ for the exchange system–environment interaction in the case of the two-qubit system. Upper panel: $\bar{I}/H_{S}$ vs. fN and *t*. Lower panel: ${\bar{I}}_{3}\left(S:B:C\right)$ as a function of *t*. (**a**,**c**) N=4. (**b**,**d**) N=5. The remaining parameters are the same as those in Figure 7.

Now, we consider the two-qubit system. The initial entangled state $|\phi_{SE_{1}}\rangle$ between the system and ancilla $E_{1}$ is prepared in the same state as Equation (22) in Section 3.1, and all the ancillas $E_{k}$ (k≥2) are initially prepared in the identical state $|\eta_{k}\rangle =|0\rangle$. Figure 10a,b shows the normalized mutual information $\bar{I}/H_{S}$ as a function of *t* and fN. Different from the single qubit system, it can be seen that there is no redundant encoding of system information and the feature of quantum Darwinism is completely lost. Additionally, at some time, $\bar{I}/H_{S}$ takes on an *S*-shaped profile, indicating an “encoding” environment (like line (c) in Figure 4) or an antiredundancy, i.e., information about *S* is encoded in the multiple environmental ancillas, and to learn about the system, one requires access to at least half of the environmental ancillas. Interestingly, we find that this “encoding” environment behavior is associated with the scrambling of information. From Figure 10c,d, it can be seen that ${\bar{I}}_{3}$ becomes negative, indicating the scrambling or delocalization of quantum information.

**Figure 11:**
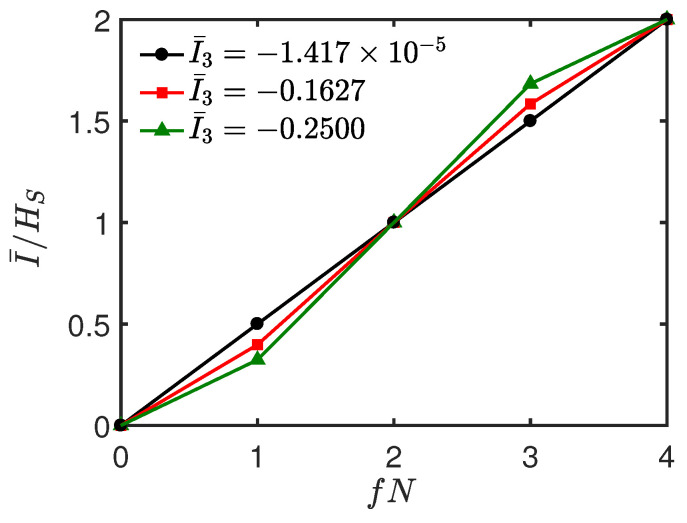
Normalized mutual information $\bar{I}/H_{S}$ as a function of fN at different *t* for the exchange system–environment interaction. The black (circles), red (squares), and green (triangles) lines correspond to *t* = 0.59, 0.70, and 0.80, respectively. All parameters are the same as those in Figure 10a.

In Figure 11, we plot the normalized mutual information $\bar{I}/H_{S}$ as a function of fN at some instants of time corresponding to different values of ${\bar{I}}_{3}$. It can be seen from Figure 11 that when $\bar{I}/H_{S}$ varies linearly with fN, the corresponding ${\bar{I}}_{3}\approx 0$ (cf. the black (circle) line in Figure 11). When ${\bar{I}}_{3}<0$ (i.e., scrambling occurs), an “encoding” environment appears, where $\bar{I}/H_{S}$ takes on an *S*-shaped profile. The larger the absolute value of the negative ${\bar{I}}_{3}$, the more pronounced the *S*-shaped profile of the curve (cf. the red (square) and green (triangle) lines in Figure 11). This is because a negative ${\bar{I}}_{3}$ with a larger absolute value means that more information about the system *S* is nonlocally stored in the joint environment fraction BC and cannot be detected by local measurement just on *B* or *C*.

In order to better understand the above phenomenon, we consider again the general model (mentioned in Section 3.1). We suppose that the joint system–environment state is initially the same as Equation (15), and after time *t*, the whole state of the system and environment evolves into

(23) $$|\Phi_{t}\rangle =\sum_{k}\alpha_{k}|\phi_{k}\rangle ⊗|\xi_{k}\rangle ,$$

where $|\xi_{k}\rangle$ are many-body entangled states of the environment. In this case, due to the presence of entanglement between the environmental ancillas, information about the system is shared among the joint environment ancillas rather than among the individual ancillas. Hence, any measurement on a small fraction of the environment may not obtain enough information about the system. Specifically, we assume that the system *S* is a qubit and the environment is composed of *N* qubits for which the evolved state $|\Phi_{t}\rangle$ is supposed to be

(24) $$|\Phi_{t}\rangle =|0\rangle ⊗|D_{N}^{\left(2\right)}\rangle +|1\rangle ⊗|D_{N}^{\left(1\right)}\rangle ,$$

where environmental state $|D_{N}^{\left(d\right)}\rangle$ is an *N*-qubit Dicke state with *d* excitations, defined as

**Figure 12:**
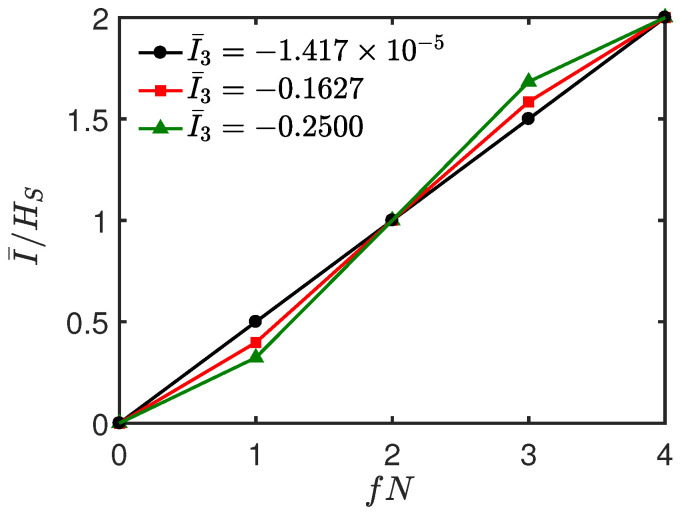
Normalized mutual information $\bar{I}/H_{S}$ as a function of fN for different *N*. The plotted curves are the results of averaging over 1000 possible environment fragments with the same *f*.

(25) |DN(d)〉=Nd−1/2∑iPi{|1〉⊗d|0〉⊗(N−d)},

where $\sum_{i}P_{i}\left\{\cdot \right\}$ denotes the sum over all possible permutations. For the state of Equation (24), we first calculate the TMI between the *S*, *B*, and *C* and obtain $I_{3}\left(S:B:C\right)<0$, which is independent of the choice of *l*. This negative TMI implies that initially localized system information is scrambled in the environment. Then, from Equation (24), in Figure 12, we plot normalized mutual information $\bar{I}/H_{S}$ as a function of environment fractions fN for different environment sizes N=5, N=6, and N=7. It can be seen that $\bar{I}/H_{S}$ takes on an *S*-shaped profile, indicating an “encoding” environment. Therefore, negative TMI is related to an “encoding” environment.

**Figure 13:**
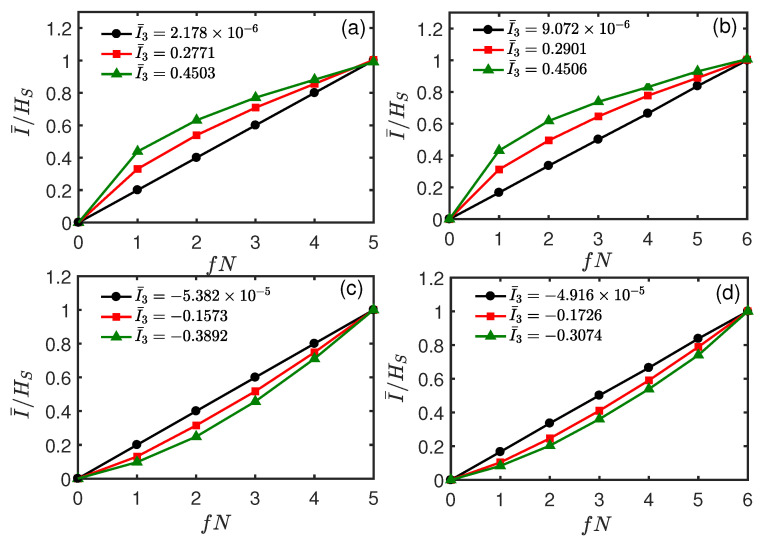
Normalized mutual information $\bar{I}/H_{S}$ as a function of fN for the exchange system–environment interaction. Upper panel: single qubit system with the same parameters except *N* as in Figure 9. Lower panel: two-qubit system with the same parameters except *N* as in Figure 10. The black (circles), red (squares), and green (triangles) lines correspond to *t* = 0.26, 0.50, and 0.80, respectively. (**a**,**c**) N=10. (**b**,**d**) N=12.

Now, we consider the above results for a larger environment with N=10 and N=12. In Figure 13, we plot $\bar{I}$ as a function of fN for different *t*, where fN varies from 0 to N/2. For the single qubit system in Figure 13a,b, we can see the redundant features of Darwinism corresponding to positive TMI, which means that information scrambling does not occur. As the positive ${\bar{I}}_{3}$ increases, redundant features of Darwinism become more and more pronounced. For the two-qubit system in Figure 13c,d, we see the behavior of an “encoding” environment, and it is related to the negative TMI. The curve with a more pronounced *S*-shaped profile coincides with the negative ${\bar{I}}_{3}$ with a larger absolute value.

Moreover, for the numerical results in this section, we emphasize the following:

(i) In the case of a single qubit system, the initial system–environment entangled state that we choose in Equation (14) is just one of the Bell states, namely, ${(|0\rangle }_{S}⊗{|0\rangle }_{E}-{|1\rangle }_{S}⊗{|1\rangle }_{E})/\sqrt{2}$. The above results are valid when it is replaced by any of the other three orthogonal entangled Bell states.
(ii) In the case of a two-qubit system, from our numerical calculations, we find that our above conclusions are valid when the initial entangled state $|\phi_{SE_{1}}\rangle$ that we choose (see Equation (22)) is replaced by the GHZ state or W state.
(iii) In the case of a two-qubit system, our results in this section are valid when the interaction Hamiltonian between $S_{1}$ and $S_{2}$, namely, Equation (3), is replaced by $H_{S_{1},S_{2}}=\epsilon \left(\sigma_{x}⊗\sigma_{x}+\sigma_{y}⊗\sigma_{y}+\sigma_{z}⊗\sigma_{z}\right)$.
(iv) As previously mentioned, to compute TMI, we divide the whole environment *E* into three nonoverlapping subsystems *B*, *C*, *D*, whose sizes (the number of the ancillas) are, respectively, given by 1, *l*, and N−l−1. Although our above numerical calculation for ${\bar{I}}_{3}\left(S:B:C\right)$ is in terms of l=2, from numerical calculation, we find that all the above results are independent of *l*.
(v) For the system–environment interactions besides Equations (5) and (6), we also consider the isotropic Heisenberg interaction (i.e., $J_{x}=J_{y}=J_{z}=1$) and anisotropic Heisenberg interaction (i.e., $J_{x}=J_{y}=1$ and $J_{z}=▵\in \left(0,1\right)$), respectively. For these two interactions, the corresponding results are qualitatively the same as those obtained from the exchange interaction.

For the above results in this paper, the key to the emergence of quantum Darwinism is the particular structure of the joint system–environment state, with a branching structure produced by the decoherence process. Each branch is perfectly correlated with a particular pointer state of the system. The subenvironments are not entangled with each other, only correlated via the system. The state of system is thus redundantly encoded in the environment itself. Different from this, when scrambling takes place, the initially localized information becomes more and more non-local and inaccessible. In this case, it will typically lead to entanglement between the subenvironments, which decreases their information storage capacity. In other words, even though the environment as a whole may still contain a redundant imprint of the state of the system, one can become inaccessible to system information only through local measurements on its small environment fragment. This is why when scrambling occurs, we do not see the Darwinistic behavior but an “encoding” environment. Quantum Darwinism and “encoding” environment behaviors are distinct ways of many-body quantum information spreading [55]. Through the TMI, we can determine whether system information is encoding redundantly in the environment, which contributes to our understanding of quantum Darwinism.

## 4. Conclusions

How the macroscopic classical world emerges from the framework of quantum mechanics has always been a topic of fundamental interest. Quantum Darwinism provides an explanation of classical objectivity from the quantum formalism, which not only regards the environment as the cause of decoherence, but also as the carrier of information about the system. The environment selects and proliferates the information of the system so that many observers can obtain the same information of the system. While information scrambling is a recent hot topic in the study of quantum many-body systems, in a process of information scrambling, initially localized information gets encoded into the global entanglement among the entire system and hence becomes inaccessible by local measurement. It seems that quantum Darwinism and information scrambling are two different research topics and have no connections. In this paper, from a new perspective and by considering the environment, is a multipartite system where we have explored the relationship between quantum Darwinism and information scrambling in the environment. The key ingredient for both quantum Darwinism and information scrambling is the information: quantum Darwinism is about the proliferation or redundant records of the information of a quantum system in the environment, while information scrambling is about how the initial local information becomes delocalized. The mutual information between the system and a subset environment has been widely used in the study of quantum Darwinism. In order to describe both quantum Darwinism and information scrambling in a unified framework, we have used TMI, which, besides OTOC, is also widely used as a probe of information scrambling. Based on a collision model, we mainly address the relation between the emergence of quantum Darwinism and information scrambling in the environment. We consider a system (one or two qubits) interacting with an ensemble of environment ancillas through two types of system–ancilla interactions, specifically, the pure dephasing interaction and exchange interaction. We find that, for both single qubit and two-qubit systems and both system–ancilla interactions, when the mutual information between the system and environmental fragment exhibits a linear increase with an increasing fragment size, TMI is zero, which can be proved in a general scenario beyond the collision model. In the case of the pure dephasing interaction for both one qubit and two-qubit systems, as TMI increases from zero, the Darwinistic behavior begins to emerge, and the larger the value of TMI, the more pronounced the Darwinistic behavior becomes. In the case of the exchange interaction, we find that the single qubit and two-qubit systems behave differently for the emergence of quantum Darwinism and scrambling. For the single qubit system, although no perfect redundant encoding appeared, the key features of quantum Darwinism still exist. Additionally, this behavior coincides with relatively small but positive TMI. However, for the two-qubit system, the “encoding” environment appears, and there is no redundant encoding of the system information; at the same time, TMI is negative, which means the information scrambling emerges. This can be explained as follows. When the TMI is negative, the system information is predominantly nonlocally shared among the environment ancillas, such that local measurement just on a small fraction of the environment cannot acquire the information of the system, leading to the loss of the quantum Darwinism feature. In contrast, when TMI is positive, more information is shared among individual environment subsystems. Thus, a measurement on a small fraction of the environment can have access to almost the same amount of information about the system, leading to the emergence of quantum Darwinism. Finally. we believe our work might shed some light on the mechanism of quantum Darwinism and stimulate more involved works in this direction.

## Data Availability

Data sharing is not applicable to this article.
